# Magnetic-Controlled Microrobot: Real-Time Detection and Tracking through Deep Learning Approaches

**DOI:** 10.3390/mi15060756

**Published:** 2024-06-05

**Authors:** Hao Li, Xin Yi, Zhaopeng Zhang, Yuan Chen

**Affiliations:** 1 Department of Mechatronics and Information Engineering, Shandong University at Weihai, Weihai 264209, China; yustlh@sdu.edu.cn; 2 Department of Mechanical Engineering, Yanbian University, Yanji 133002, China; yxin0912@gmail.com (X.Y.); rocman215@outlook.com (Z.Z.)

**Keywords:** microrobots, deep learning, target detection, real-time imaging

## Abstract

As one of the most significant research topics in robotics, microrobots hold great promise in biomedicine for applications such as targeted diagnosis, targeted drug delivery, and minimally invasive treatment. This paper proposes an enhanced YOLOv5 (You Only Look Once version 5) microrobot detection and tracking system (MDTS), incorporating a visual tracking algorithm to elevate the precision of small-target detection and tracking. The improved YOLOv5 network structure is used to take magnetic bodies with sizes of 3 mm and 1 mm and a magnetic microrobot with a length of 2 mm as the pretraining targets, and the training weight model is used to obtain the position information and motion information of the microrobot in real time. The experimental results show that the accuracy of the improved network model for magnetic bodies with a size of 3 mm is 95.81%, representing an increase of 2.1%; for magnetic bodies with a size of 1 mm, the accuracy is 91.03%, representing an increase of 1.33%; and for microrobots with a length of 2 mm, the accuracy is 91.7%, representing an increase of 1.5%. The combination of the improved YOLOv5 network model and the vision algorithm can effectively realize the real-time detection and tracking of magnetically controlled microrobots. Finally, 2D and 3D detection and tracking experiments relating to microrobots are designed to verify the robustness and effectiveness of the system, which provides strong support for the operation and control of microrobots in an in vivo environment.

## 1. Introduction

In the frontier of science in the 21st century, microrobots have undoubtedly emerged as one of the most prominent and captivating research fields [1]. In recent years, microrobots have shown great advantages in the field of biomedical applications. With the development of microrobots, various aspects such as their driving methods [2,3,4,5,6], recognition and tracking [7,8], biosafety [9], targeted drug delivery methods [10,11,12], and multi-functional integration [13,14] have attracted widespread attention. For example, a magnetically driven rotary ablation catheter robot [15] was employed to remove calcified deposits from arterial stenosis and occlusion. A cylindrical microrobot [6] driven by a permanent magnet array was designed to continuously penetrate through and navigate around the soft tissue. A wireless modular capsule robot [14] was utilized to accomplish the tasks of reorganization, navigation, and separation within the gastric environment, effectively addressing the size-related challenges encountered by multifunctional capsule robots.

While the application of microrobots in the medical domain has progressed steadily, practical implementation remains a considerable challenge, with numerous outstanding issues. These challenges include the difficulty of driver or power installation due to the reduced size of microrobots, material safety concerns, real-time position and environment observation limitations, and other related issues. Among these challenges, the recognition and tracking of microrobots have gradually garnered attention from many researchers. Given the micro–nano scale of microrobots and the inherent complexity of their operating environments, substantial global research efforts have been dedicated to addressing the issues of positioning and tracking. In order to achieve stable tracking in pulsating fluid, Li [4] designed a tracking strategy both within and perpendicular to the image plane. A strategy based on iterative statistics was proposed to obtain the position and attitude of the robot from ultrasonic images. From the perspective of imaging, Bappy [7] proposed a method of using haze removal image enhancement as a pre-processing method and a multi-level threshold as a post-processing method to realize the automatic reconstruction of a 3D vascular model. Nguyen [8] proposed a real-time position and spatial orientation tracking method for millimeter intravascular microrobots based on principal component analysis and X-ray reconstruction. In addition, due to the high imaging contrast of biological tissue, magnetic resonance imaging [16] is widely used in the real-time tracking and driving of microrobots. In recent years, deep learning technology [17] has become quite mature in the computer field, and deep learning has been widely used in computer vision-related tasks, such as target detection [18,19], semantic segmentation [20], target classification [21], and so on. Although deep learning technology has been widely explored and applied in other fields, such research on microrobots is relatively scarce. Consequently, researchers have initiated investigations into leveraging deep learning technology within the field of microrobots. Currently, this technology is still in a relatively early stage and requires further in-depth research and development. Meitin Sitti [22] exemplified the application of deep learning technology in an endoscopic capsule robot, proposing a localization method grounded in endoscope camera information and multi-sensor fusion. Karim Botros’ team [23] proposed a chain-like magnetic microsphere robot target detection and tracking system based on ultrasound imaging. This method uses CNN neural networks in deep learning technology to estimate the position of the microrobot in real time. Experiments show that the system can perform the high-precision real-time detection and tracking of spherical microrobots with a diameter of about 500 µm in dynamic environments. The results show that the system can detect up to 95% of spherical microrobots. ETH Zurich [24] proposed a machine learning-based magnetic control microrobot position control method that achieves position control through the gradient field generated by electromagnetic coils.

**Figure 1:**
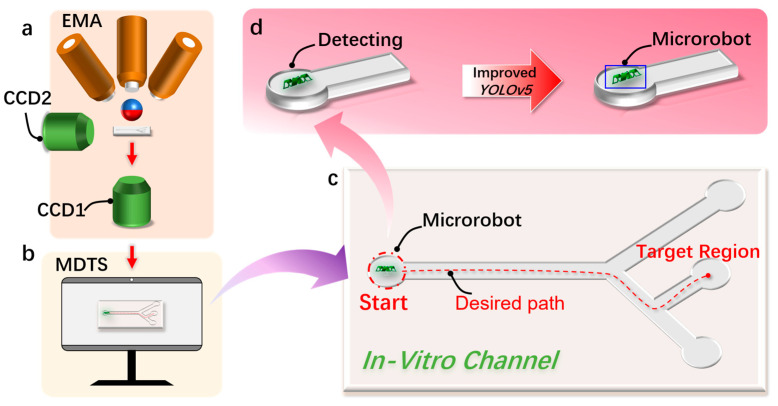
The detection and tracking schematic diagram of the magnetically controlled micro-medical robot based on deep learning. (**a**) A schematic diagram of the electromagnetic drive (EMA) system that drives the magnetically controlled microrobot to generate motion in the region of interest (ROI). (**b**) This is used to realize the schematic diagram of the microrobot target detection and motion tracking (MDTS) system. (**c**) The microrobot moves to the target position in the external orbit driven by the EMA system and uses MDTS to detect and track the trajectory of the microrobot. (**d**) Using the improved YOLOv5 target detection algorithm based on deep learning to realize the detection of the microrobot.

In this paper, combined with deep learning technology, a detection and tracking method of a magnetically controlled microrobot based on the YOLOv5 target detection algorithm is proposed, aiming to achieve the real-time recognition and tracking of a magnetically controlled microrobot in vascular environments (Figure 1). The microrobot is driven by electromagnetic means. During its movement, real-time footage collected by the camera is input into the microrobot detection and tracking system. The combination of the improved YOLOv5 target detection algorithm and visual algorithms enables the real-time detection and tracking of the magnetically controlled microrobot. In addition, based on the parameters of the human hepatic vein, we designed two-dimensional and three-dimensional epoxy resin vascular models for microrobot intravascular tracking experiments. Through the experimental validation, we confirm the effectiveness of the proposed approach, providing valuable insights for subsequent animal or clinical trials.

## 2. Materials and Methods

### 2.1. Target Tracking Algorithm

#### 2.1.1. Monocular Vision Algorithm

**Figure 2:**
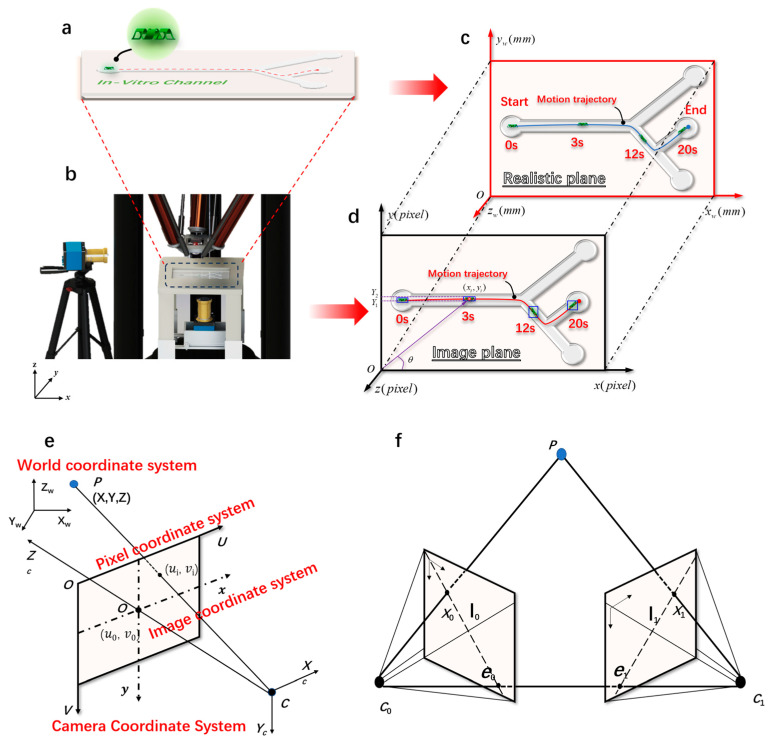
The imaging principle of magnetically controlled microrobots based on a binocular vision algorithm. (**a**) Customized microrobot motion trajectory. (**b**) Magnetically controlled binocular imaging and tracking experimental device. (**c**) A real plane diagram of the monocular target tracking algorithm. (**d**) An imaging plane diagram of the monocular target tracking algorithm. (**e**) The geometric model of camera imaging and the spatial distribution map of the four coordinate systems established. (**f**) The polar geometry of two corresponding camera images.

The monocular vision algorithm employed in this study calculated the world coordinates of the target based on its pixel coordinates. It utilized image information captured by the camera, matched the size information of the microrobot with the image information obtained through the camera, and finally obtained the length of each pixel unit. This allowed for the calculation of the microrobot’s real-time position and velocity. Compared with the traditional binocular or trinocular system, monocular vision offers advantages such as system simplicity, low construction cost, and ease of use and maintenance. Figure S2 depicts the imaging principle of a monocular camera. The calculation formula for monocular imaging is given by Equation (1):

(1) f=(p⋅D)/w

where *f* is the focal length of the camera (mm), *p* is the pixel size of the magnetic body in the imaging plane, *D* is the actual distance from the camera’s optical center to the magnetic body, and *w* is the actual size of the magnetically controlled microrobot. By placing the camera at the vertical bottom of the experimental platform, we can obtain a real-time image of the region of interest. Figure 2b depicts the camera imaging experimental platform. Figure 2c,d depict the transformation of the imaging system of the microrobot from a pixel coordinate system to a world coordinate system. The pixel coordinates and world coordinates of microrobots are represented as follows:

(2) $$\begin{array}{c} h=\left[\begin{array}{c} x_{i}\\ y_{i} \end{array}\right] \end{array}$$

(3) $$\begin{array}{c} H=\left[\begin{array}{c} x_{w}\\ y_{w} \end{array}\right] \end{array}$$

where *x_i_* and *y_i_* represent the current coordinate information of the microrobot in the pixel coordinate system and *x_w_* and *y_w_* represent the current coordinate information of the microrobot in the world coordinate system. Through the pixel coordinate point of the current frame of the microrobot and the pixel coordinate point of the previous frame, the pixel distance of each frame of the microrobot can be calculated as follows:

(4) $$\begin{array}{c} l_{p}=\sqrt{{\left(x_{i}-x_{i-1}\right)}^{2}+{\left(y_{i}-y_{i-1}\right)}^{2}} \end{array}$$

where *l_p_* represents the pixel distance of each frame of motion and *x_i−_*_1_ and *y_i−_*_1_ represent the pixel coordinate information of the last frame of the target. By accumulating the pixel distance of each frame of the microrobot, the pixel distance of the whole motion stage of the microrobot is obtained and calculated as follows:

(5) $$\begin{array}{c} L_{p}=\overset{n}{\underset{i=0}{\sum }}l_{p} \end{array}$$

where *L_p_* represents the pixel distance of the microrobot in the whole motion stage. We used the improved YOLOv5 to train the magnetic robot, and the weight model was employed to identify and track the magnetic microrobot. Through the identified target detection box, we obtained the pixel coordinate information of the target:

(6) $$\begin{array}{c} P_{LT}=P_{2}=\left[\begin{array}{c} X_{2}=P_{2}\left[0\right]\\ Y_{2}=P_{2}\left[1\right] \end{array}\right] \end{array}$$

(7) $$\begin{array}{c} P_{RB}=P_{1}=\left[\begin{array}{c} X_{1}=P_{1}\left[0\right]\\ Y_{1}=P_{1}\left[1\right] \end{array}\right] \end{array}$$

where *P_LT_* and *P_RT_* represent the pixel coordinate points of the upper left corner and the lower right corner corresponding to the detected microrobot detection box, respectively. Through the information of these two coordinate points, the pixel size of the target can be calculated. The formula is as follows:

(8) $$\begin{array}{c} ∆p=Y_{2}-Y_{1}=P_{2}\left[1\right]-P_{1}\left[1\right] \end{array}$$

The target is in the pixel coordinate system: the component along the *x* direction and the *y* direction, and the component along the *U* direction and the *V* direction in the world coordinate system. The calculation formula is as follows:

(9) $$\begin{array}{c} pixel:\left\{\begin{array}{c} x=\left|h\right|\cdot \cos\theta =x_{i}\\ y=\left|h\right|\cdot \sin\theta =y_{i} \end{array}\right. \end{array}$$

(10) $$\begin{array}{c} world:\left\{\begin{array}{c} U=\left|H\right|\cdot \cos\theta =x_{w}\\ V=\left|H\right|\cdot \sin\theta =y_{w} \end{array}\right. \end{array}$$

where *θ* is expressed as the angle between the target and the positive direction of *x* (0–90°). Through the above formula, the coordinates of the target in the world coordinate system can be calculated as follows:

(11) $$\begin{array}{c} \left\{\begin{array}{c} x_{w}=\frac{\left|H\right|\cdot x_{i}}{\left|h\right|}=\frac{x_{i}\cdot w}{P_{2}\left[1\right]-P_{1}\left[1\right]}\\ y_{w}=\frac{\left|H\right|\cdot y_{i}}{\left|h\right|}=\frac{y_{i}\cdot w}{P_{2}\left[1\right]-P_{1}\left[1\right]} \end{array}\right. \end{array}$$

#### 2.1.2. Binocular Stereo Vision Algorithm

Although monocular vision has the advantages of a simple system and low construction cost, it cannot obtain the three-dimensional (3D) world coordinates of points through a single camera because the coordinates obtained by monocular cameras lack dimensional information, namely depth information. Given the complex and irregular 3D environment within the human body, microrobots need to move and reach designated target points in such an irregular 3D environment. This requires obtaining depth information of 3D points in the in vitro mobility performance test, which was achieved by incorporating another camera to form binocular stereo vision. The depth information of the 3D point was calculated based on their imaging coordinates in the two cameras, thereby obtaining the 3D coordinate information of the target point. For specific principles and operation procedures, please refer to the Supplementary Information (S1).

To convert the 2D coordinates obtained from the camera imaging into 3D coordinates, we performed a conversion between different coordinate systems. This mainly involved the conversion between the pixel coordinate system, the image coordinate system, the camera coordinate system, and the world coordinate system, as shown in Figure 2e.

The principle of binocular stereo vision is based on the parallax principle, a method for obtaining 3D geometric information of objects from multiple images. In the machine vision system, binocular vision generally involves two cameras capturing two digital images of the surrounding scenery from different angles at the same time. Based on the parallax principle, it is possible to calculate the 3D geometric information of the object, reconstructing the 3D shape and position of the surrounding scenery. Figure S4 shows a heads-up binocular stereo imaging schematic diagram. The projection points *P*_0_ (*x*_0_, *y*_0_) and *P*_1_ (*x*_1_, *y*_1_) of the 3D target to be detected in the space were captured by the left and right cameras simultaneously. To facilitate calculation, a 2D model of binocular stereo vision was established, and according to the principle of triangulation, it can be deduced as follows:

(12) $$\left\{\begin{array}{c} X=\frac{B\cdot (x_{0}-u_{0})}{x_{0}-x_{1}}\\ Y=\frac{B\cdot (y_{0}-v_{0})}{x_{0}-x_{1}}\\ Z=\frac{B\cdot f}{x_{0}-x_{1}} \end{array}\right.$$

The parallax disparity = *x*_0_ − *x*_1_, and *B* is the distance between the optical centers C0 and C1 of the two cameras, also known as the baseline length (mm).

During the two-dimensional real-time detection and tracking experiment of the microrobot, the bottom camera was used to collect real-time motion videos of the micro-robot. During the three-dimensional tracking experiment, the calibrated bottom camera was used to collect real-time images of the bottom of the microrobot, and the side camera was used to collect real-time images of the side of the microrobot. The right image shows the real-time detection and tracking of the microrobot.

### 2.2. Detection Model of Microrobot

#### 2.2.1. Improved YOLOv5 Network Model

**Figure 3:**
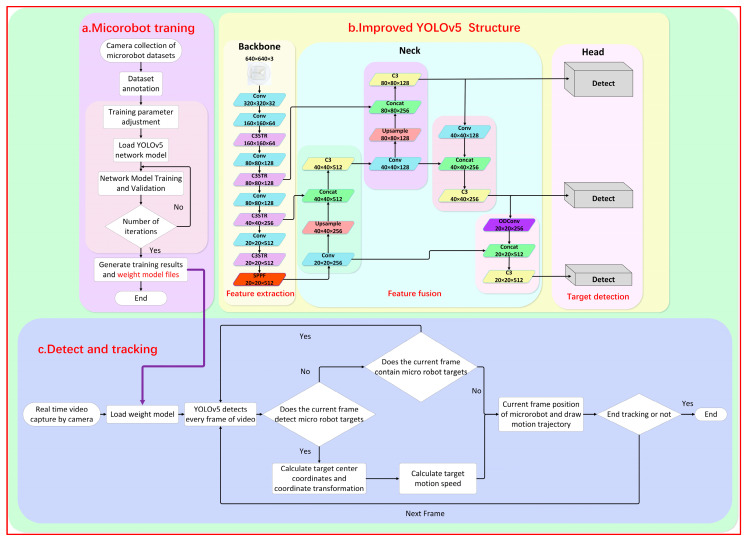
A schematic diagram of the imaging and tracking principle of the YOLOv5 magnetically controlled microrobot based on deep learning. (**a**) The improvement of the YOLOv5 network framework and the training diagram of the magnetically controlled microrobot model. (**b**) Loading the trained robot weight model file into the tracking phase to realize the detection and tracking of the experimental microrobot. (**c**) The real-time detection and tracking process of the microrobot.

During the detection experiment, it was observed that the original YOLOv5 model faced challenges in detecting the magnetic microrobot due to the imaging size range being between 10 and 40 pixels. This led to issues such as missed detections, false detections, and low detection accuracy. To enhance the original model’s ability to detect small-size targets and improve the detection efficiency, this paper proposed an improved YOLOv5 network model, as shown in Figure 3.

The background information of the dataset collected in this experiment was monotonous. The microrobot to be detected was only millimeters in size (1–2 mm in length) and thus blended with the environment, making it difficult to distinguish the magnetic body and increasing the difficulty of extracting feature information. The classical image convolution compression (convolutional neural network) operation is often used in the backbone part, which further loses a large amount of feature information, leading to a decrease in target detection accuracy or even the failure of target recognition. Therefore, this paper embedded the Swin Transformer [25] module into the backbone network C3 module, forming a new C3STR module. With the help of the Swin Transformer module, the feature extraction capability for small targets was enhanced, and the loss of feature information was reduced. Figure S1b shows the improved C3STR module.

The Swin Transformer module consists of two multilayer perceptrons (MLPs), a window attention module (window multi-head self-attention, W-MSA), a sliding-window multi-attention module (shifted window multi-head self-attention, SW-MSA), and four normalized layers, as shown in Figure S1a. Compared with the traditional convolution model, the Swin Transformer adopts a hierarchical and parallel method to process the feature information of the images. It performs compression and convolutional feature extraction on images simultaneously. The average processing speed improved from 1.5 s before the enhancement to 0.45 s after the improvement, reducing the model’s computation time and improving the efficiency of feature extraction in the backbone network.

In the final feature fusion section, we introduced an ODConv module [26]. The classic Conv is stacked from multiple convolutional layers and predefined feature connection layers, with spatial dimensions having invariance, limiting the receptive field of the convolutional layers. Compared to traditional Conv, ODConv incorporates a multi-dimensional attention mechanism.

The distinctive feature is that this multi-dimensional attention mechanism uses a parallel strategy, learning across four dimensions of kernel space: the spatial dimension, the input channel dimension, the output channel dimension, and the convolutional kernel space dimension. This enriches the extraction of feature information from the upper and lower images, enhancing the entire network’s feature extraction capability. Additionally, the ODConv module introduces an adaptive adjustment module to adaptively adjust the weights of the convolution kernel. This allows the model to automatically adjust the receptive field and weight of the convolutional kernel based on the local feature information of targets of different sizes, improving the accuracy and robustness of target detection. The structure of ODConv is shown in Figure S1c.

#### 2.2.2. Fabrication of Microrobots

**Figure 4:**
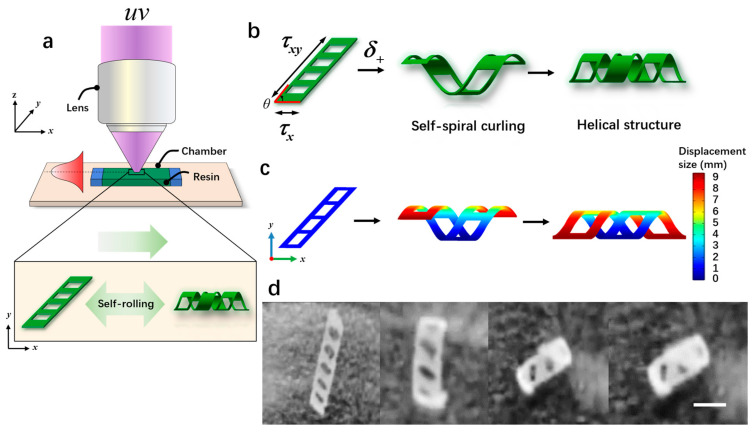
The fabrication and the self-curling principle of magnetically controlled microrobots. (**a**) The schematic diagram of the light-curing process of the microrobots. (**b**) The self-curling principle of the microrobots. (**c**) The self-curling simulation image of the microrobots. (**d**) The self-curling optical microscopic image of the microrobots. Scale bar: 2 mm.

Microrobots were manufactured from direct patterning and visual optical systems, as shown in Figure 4a. The fabrication process of the microrobots was as follows: First, we took two transparent glass slides with an area of 24 × 24 mm and 20 × 20 mm, and then double-sided tape (thickness: 100 mm) was sandwiched between them to form a microchamber. Next, a biocompatible solution was prepared by mixing 50 wt% e-dent 400 and 50 wt% MMP using a Thinky Mixer (Nano Tech, Inc., Daejeon, Republic of Korea) at a speed of 2000 rpm for 30 min. Once the microchamber was prepared, a syringe was used to inject the biocompatible solution directly into the microchamber using capillary force. Subsequently, the microchamber was placed in a sample box and polymerization was carried out under ultraviolet light (λ = 365 nm) for 3.2 s. Previous studies [27] have demonstrated that the curvature of microrobots is maximized when the ratio of the soft layer to the hard layer is 8:2, resulting in a more stable self-curling structure. Exposed samples were placed in a culture dish filled with isopropanol (IPA) and covered with aluminum foil for 2 h. Afterward, the glass slide and tape were taken out, and direct cleaning with isopropanol was performed to remove the unpolymerized ink. The patterned film remained on the lid glass. Finally, a few minutes later, the patterned film self-separated and curled relative to the initial design, as shown in Figure 4d and Supplementary Video S1. When the oblique film (θ < 90°) was exposed to proton stimulation (δ+), torque was generated due to the different lengths along the *x*-axis and *y*-axis, resulting in asymmetric folding. This folding caused the film to form a spiral structure (Figure 4b). Figure 4c illustrates the simulated rolling phenomenon occurring over time under exposure to proton stimulation (δ+). Initially, the rolling angle of the film was 0° (φ); as time passed, the rolling angle (φ) increased to 360°, finally forming a spiral structure. In addition, the air hole in the design played a crucial role in the generation of the spiral structures. If there were no air holes, the generated torque would tilt, resulting in the formation of a cylindrical structure.

#### 2.2.3. Dataset Making and Evaluation Index of Microrobot

The datasets used in this paper were collected by means of self-selecting cameras and other equipment, including 1500 pictures of magnetic bodies 3 mm in size, 1500 pictures of magnetic bodies 1 mm in size, and 3000 datasets of microrobots with a length of 2 mm. Before training based on the pictures, the image annotation tool Labelimg v3.9 was used to label the target pictures of different sizes, and the annotated dataset was divided into a training set and verification set according to the proportion of 8:2 to ensure that the model could learn all kinds of features. In this paper, the accuracy (precision), recall rate (recall), and average precision mean (mean average precision) were used as evaluation indicators. The calculation formulas for these indicators are as follows:

(13) $$P=\frac{TP}{TP+FP}$$

(14) $$R=\frac{TP}{TP+FN}$$

(15) $$mAP=\frac{\sum_{i=1}^{k}AP_{i}}{k}$$

where *TP* represents the number of positive samples predicted as positive, *FP* represents the number of negative samples predicted as positive, *FN* represents the number of positive samples predicted as negative, *k* represents the number of categories, and *AP_i_* represents the average accuracy at time *i* in the area enclosed by the P-R curve and the *x*-axis and *y*-axis.

The operating system used in this experiment was Windows10, the programming language used was Python v3.9, the editor used was PyCharm 2023, the deep learning framework employed was Pytorchv1.13, CUDA version 11.6, the CPU used was Intel (R) Core (TM) i5-13600KF3.50GHz, and the GPU employed was NVIDIA GeForceRTX3060.

#### 2.2.4. Driving System of Magnetically Controlled Microrobot

The EMA (electrical magnetic actuation) system consisted of three electromagnetic coils with copper rods, and at the end of each coil, there was a permanent magnet ball with a diameter of 30 mm. This combination of permanent magnet and electromagnet components was used for the testing of the programmatical control of 2D and 3D motion performance of the microrobot in orbit, as shown in Figure 2b. The movable range of the permanent magnet in the drive system is a cylindrical space with a diameter of 27 cm and a height of 66 cm. The coil assembly is made of pure aluminum and contains a coil wound with 350 turns of 1 mm diameter double-layer copper wire. Below the coil is a freely rotating neodymium–iron–boron (NdFeB) magnetic sphere with a surface magnetic induction intensity of 5000 Gs. The orientation of the magnetic sphere is controlled by the magnetic field generated by the electromagnetic coils, thereby controlling the posture of the microrobot. The electromagnetic coils are powered by an Aideck IT6942A (Luoyang Hengkai Technology Co., Ltd., Luoyang, China) programmable DC power supply. A LabVIEW control program monitors the experimental power supply and determines the output current of each power supply based on the direction of the end effector’s movement in the triangular structure. By adjusting this, the direction of the magnetic field at the end is changed to control the orientation of the magnetic sphere. The drive system is controlled by a NET_AMC3XER V1.1 three-axis motion control card, three C-DR42A stepper motor drivers, and three fulsun42 stepper motors as power sources. The step angle of the stepper motor is 1.8°, the torque is 420 mN·m, and the maximum operating rate is 2000 PPS. The LabVIEW program manages the operation of the stepper motors. By inputting the motion coordinates of the end structure on the front panel of the LabVIEW control program, the LabVIEW control program sends the number of motion pulses and directions for each motor to the NET_AMC3XER V1.1, which generates the corresponding pulses to the stepper motor drivers to determine the direction and number of steps. A fully transparent resin fluid channel, manufactured via UV-cured 3D printing, was placed in the workspace of the EMA system. A binocular camera was placed below the EMA system to detect and track the motion of the microrobot.

## 3. Results

### 3.1. Microrobot Comparative Experiment

**Figure 5:**
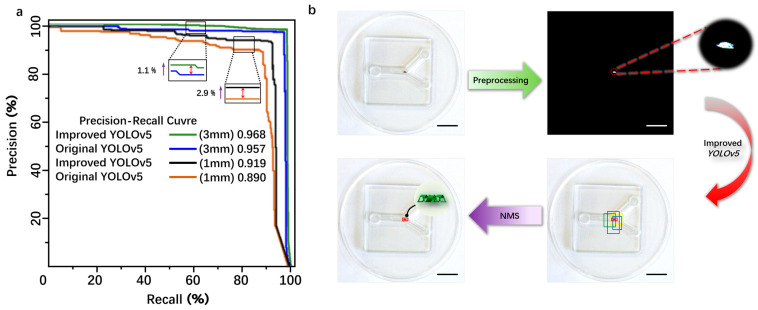
(**a**) The comparative experimental analysis of the improved YOLOv5 and the traditional YOLOv5 detection. (**b**) The schematic diagram of the detection principle of the magnetically controlled microrobot based on YOLOv5. Scale bar: 10 mm.

The original YOLOv5 and the improved YOLOv5 were trained and validated using the same dataset. The specific experimental results are shown in Table 1. As indicated by Table 1, for the 3 mm magnetic body target, the comparative evaluation results show that the improved accuracy (precision, P) is 95.81%, an increase of 2.1%, the recall rate (R) is 92.33%, an increase of 2.12%, and the mean average precision (mAP) is 96.8%, an increase of 1.1%. For the 1 mm magnetic body target, the comparative evaluation results show that the improved precision (P) is 91.03%, an increase of 1.33%, the recall rate (R) is 90.30%, an increase of 0.57%, and the mAP is 91.9%, an increase of 2.9%. For the 2 mm microrobot target, the comparative evaluation results show that the improved precision (P) is 91.70%, an increase of 1.5%, the recall rate (R) is 94.30%, an increase of 1.7%, and the mAP is 96.2%, an increase of 1.7%. Overall, the improved YOLOv5 model proposed in this paper exhibits superior detection performance compared to the original model. The introduction of C3STR and ODConv contributes to enhanced detection accuracy for small targets, addressing the original model’s deficiencies in missed detections and false positives. The average processing speed is reduced from 1.5 s before improvement to 0.45 s after improvement, reducing the computation time and meeting real-time detection requirements. The P–R curve was obtained by using the magnetic bodies with sizes of 3 mm and 1 mm as the dataset shows in Figure 5a.

From Figure 5a, it can be seen that the area below the P–R curve of the improved YOLOv5 model is slightly larger than that of the original YOLOv5 model, indicating that the improved YOLOv5 model has a higher average precision (AP) than the original model. The detection process of the microrobot target by YOLOv5 is as follows: First, the camera captures images of the microrobot and preprocesses them by changing the image size to the YOLOv5 detection size of 640 × 640 × 3 for subsequent detection. Then, the image is binarized to reduce the interference from background information. Next, the image is input into the improved YOLOv5 neural network framework for detection. Finally, after non-maximum suppression (NMS) processing, detection boxes with low intersection over union (IOU) values are removed, leaving only the detection box with the maximum IOU value to achieve the detection of the microrobot. The IOU threshold for this experiment is set to 0.7 to filter out detection boxes with low IOU values during the detection process, ensuring that the detection boxes can match the target to the maximum extent, as shown in Figure 5b.

### 3.2. Real-Time Detection and Tracking of Microrobot

**Figure 6:**
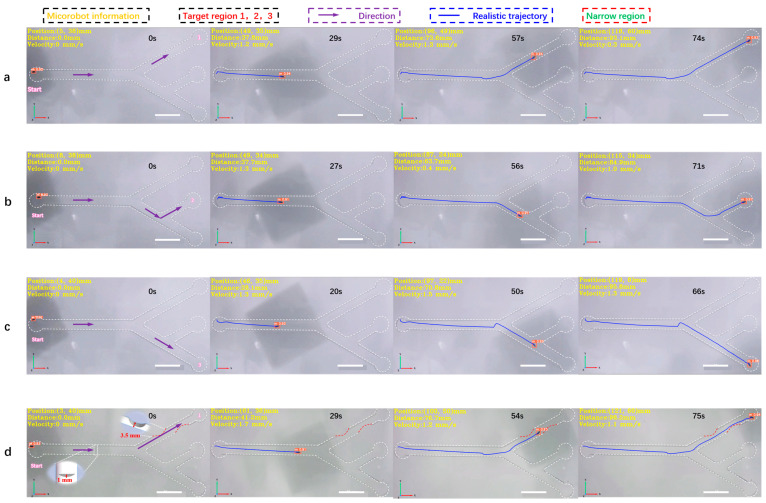
The 2D detection and tracking experiment of the magnetically controlled microrobot based on YOLOv5. (**a**) The microrobot moves to target area 1. (**b**) The microrobot moves to target area 2. (**c**) The microrobot moves to target area 3. (**d**) The microrobot passes through an obstacle with a height of 1 mm, through two narrow areas, the width of which is 3.5 mm, and moves to target area 1 to simulate the thrombus deposited on the inner wall of blood vessels. In this experiment, the main diameter of the vascular model was 6 mm and the diameter of the branch was 5 mm. All parameters simulate the establishment of human hepatic veins. Scale bar: 10 mm.

In this experiment, an electromagnetic driving method was employed. In order to achieve the real-time detection and tracking of microrobots, a microrobot detection and tracking (MDTS) system was designed. The flowchart of the microrobot detection and tracking system is shown in Figure 2c. Cameras (HDC60, f-4-12 mm 1600 w Pixel, MOKOSE, Shenzhen, China) were placed at the bottom and side of the workspace, respectively, for the real-time imaging of microrobots. The video sequence captured by the cameras was inserted into MDTS, and a series of preprocessing steps were applied to the real-time video. The video was resized proportionally, centered, and padded with excess parts for subsequent detection. After the preprocessing, the microrobot weight model trained by the improved YOLOv5 was loaded into MDTS, and the training process presented in Figure 3a was followed. The preprocessed video frames were input into the MDTS for detection. If the system detects the microrobot target, it calculates the centroid coordinates of the target, transforming them into world coordinates using the target tracking algorithm, and computes the microrobot’s motion speed in each frame. This process yields the precise position of the microrobot in the current frame, and the trajectory of the microrobot is plotted. The blue curve in Figure 6 represents the motion trajectory of the microrobot. If tracking is not complete, and the camera is still capturing video, the next frame of the detection and tracking is performed.

In this experiment, a 2D vascular model (Figure S5a) was established according to the parameters of human hepatic veins [28], with an overall size of 150 mm × 75 mm. The main diameter was 6 mm, while the bifurcation diameter was 5 mm. Three target areas, 1, 2, and 3, were set at the top, middle, and bottom ends of the vascular model, respectively. The pipeline was filled with DI water, as shown in Figure 6. Under the joint drive of the electromagnetic coil and magnetic ball, the microrobot moved from the leftmost point of the screen to target area 1. The MDTS system achieved full-process detection and tracking of the microrobot, with an average detection accuracy of 0.91 and no false positives or misses. The calculated average speed of the microrobot was 1.3 mm/s. From the plotted motion trajectory, it can be observed that the movement of the microrobot is smooth, without sudden shifts caused by excessive magnetic force, as shown in Figure 6a and Supplementary Video S2. Similarly, when the microrobot moved from the leftmost point to target area 2, the average detection accuracy was 0.92, and there were no false positives or misses. The calculated average speed of the microrobot was 1.3 mm/s. The motion trajectory shows that the microrobot’s movement is still smooth, without sudden shifts due to the excessive magnetic force (Figure 6b, Supplementary Video S2). When the microrobot moved from the leftmost point to target area 3, there were no false positives or misses, as shown in Figure 6c, and Supplementary Video S2. Finally, narrow and obstructed areas were added to the orbit to simulate thrombosis in the inner wall of blood vessels. A 3D-printed thrombus model was placed in the pipeline, with a narrow area width of 2.8 mm, much smaller than the normal diameter of the pipeline. The microrobot passed through the narrow area under electromagnetic drive, slowing down to an average speed of around 0.3 mm/s. The MDTS system still did not show any false positives or misses. Supplementary Video S3 shows that the microrobot experienced a brief pause and unstable motion when passing through the narrow area. The reason for this phenomenon may be the non-smooth surface of the 3D-printed thrombus model, leading to increased resistance when the microrobot comes into contact with the thrombus surface. Another possibility is that the small bubbles (<1 mm) remaining on the inner wall of the track come into contact with the microrobot, increasing the resistance during the movement. To address this, a customized transparent track model with thrombosis and obstacles (Figure S5b) was later developed using epoxy resin photocuring. This reduced the friction resistance on the surface of the model. The obstacle height was set at 1 mm, while the width of the two narrow areas was 3.5 mm. The motion process was smooth and good detection accuracy was obtained, as shown in Figure 6d and Supplementary Video S3.

**Figure 7:**
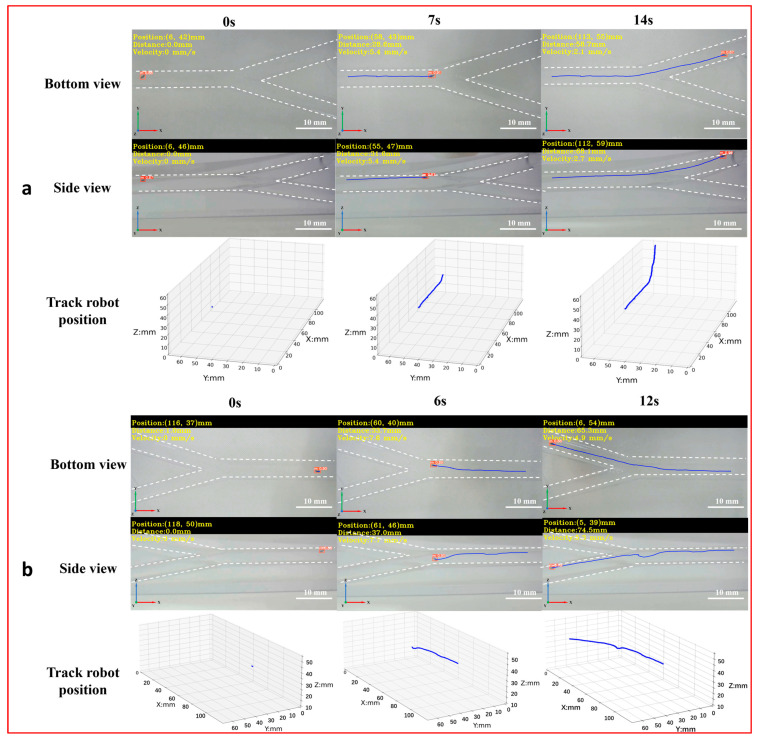
The 3D detection and tracking experiment of the magnetically controlled microrobot based on YOLOv5. (**a**) The microrobot moves to bifurcation 1: the left image is the side and top plane of the microrobot captured by the camera, and the right image is the 3D trajectory of the microrobot. (**b**) The microrobot moves to bifurcation 1: the left image is the side and top plane of the microrobot captured by the camera, and the right image is the 3D trajectory of the microrobot. Scale bar: 10 mm.

To achieve the 3D detection and tracking of the microrobot by MDTS, a 3D vascular model (Figure S5c) was designed with dimensions of 150 mm × 75 mm × 53 mm, a diameter of 6 mm, and two bifurcations labeled as bifurcation 1 and bifurcation 2 (downward). The real-time motion images of the microrobot captured by the side and bottom cameras were input into the MDTS for detection and tracking. The system calculated the 3D position information of the microrobot and plotted its 3D motion trajectory in the vascular model (Figure 7, Supplement Video S4). From the real-time 3D trajectory of the microrobot drawn by MDTS, it can be observed that when the microrobot moved to bifurcation 1, there was a brief undulating motion. This was because the target area of the microrobot was inclined downward at bifurcation 1. The distance between the electromagnetic drive end and the microrobot changed constantly, causing the distance between the microrobot and the end of the electromagnetic drive system to increase. As a result, the influence of electromagnetic drive on the microrobot decreased, leading to a brief undulating motion. When the target area of the microrobot was at bifurcation 2, this phenomenon did not occur. This is because bifurcation 2 was inclined upward, and as the microrobot moved, the distance between it and the electromagnetic drive end gradually decreased, always remaining within a controllable range.

## 4. Discussion

In this study, we propose a real-time detection and tracking system for magnetically controlled microrobots based on deep learning. We replace traditional convolution modules with C3STR modules in the backbone network and introduce the ODConv module during the feature fusion stage, specifically addressing targets such as microrobots with sizes of less than 40 pixels. This enriches the extraction of feature information from the upper and lower images, enhancing the overall feature extraction capability of the network. The improved YOLOv5 network model achieves an accuracy of 95.81% for recognizing 3 mm magnetic bodies, representing an improvement of 2.1%. For 1 mm magnetic bodies, the recognition accuracy is 91.03%, representing an improvement of 1.33%. The recognition confidence for magnetically controlled microrobots reaches 0.91, validating the fact that the improved YOLOv5 network model can achieve real-time detection and recognition throughout all stages of magnetically controlled microrobots without missing or misidentifying. The combination of the improved YOLOv5 network model with visual algorithms effectively realizes the real-time detection and tracking of magnetically controlled microrobots. Two- and three-dimensional tracking experiments were conducted, successfully obtaining position information, motion trajectories, motion distances, and speed information of microrobots during the programmable magnetic control process.

During the experiment, challenges arose in the detection and tracking of microrobots due to interference from lighting and the electromagnetic driving system in the captured images, especially with one camera positioned at the bottom of the experimental track (Figure 2b). To address this issue, a 3D-printed white background model with dimensions of 200 mm × 200 mm was created and placed on top of the experimental track to reduce interference and enhance detection accuracy. When designing the 3D tracking experiments, the initially customized 3D vascular model served as a navigation track and was positioned inside the model. Due to limitations in the light-curing printing equipment, the prepared inner surface of the track could not achieve absolute smoothness and polishing, resulting in a relatively poor imaging effect for microrobots. Consequently, we redesigned the 3D vascular model, placing the track inside a transparent groove with dimensions of 150 mm × 75 mm × 53 mm to address the issue of uneven surfaces within the track. When injecting DI water into the groove, the rapid injection speed led to the swift filling of the track, preventing residual air from escaping, and resulting in the formation of bubbles. Even bubbles with a diameter of 1 mm could interfere with the detection of microrobots when observed by the camera. To address these issues, the solution involved controlled DI water injection using a modified syringe and allowing the experimental track filled with DI water to stand for 30 min. This approach maximized the removal of air bubbles within the track, minimizing their impact on the imaging of the microrobots. Initially, our experimental design involved placing two cameras parallel to each other at the bottom of the track. However, due to the imaging range of the cameras being limited to 200 mm × 200 mm, the captured images displayed an incomplete view of the track. As a solution, adjustments were made to the camera’s focal length and the distance between the camera and the track. During the adjustment, it was discovered that to capture the complete track information on the imaging plane, the track needed to be elevated. However, the movement space of the entire electromagnetic driving system was limited. Elevating the track would compress the movement space of the electromagnetic coils, preventing its movement. To address this issue, the experiment was optimized by placing one camera on the side of the track and another at the bottom (Figure 2b). This solution resolved the issue of an incomplete track display in the captured images. During the detection and tracking of microrobots, no instances of missed detections or false detection were observed, and the calculated precision of the microrobot’s positions remained satisfactory.

In the future, we plan to introduce obstacles into the 3D vascular model to validate MDTS for detecting and tracking microrobots in a 3D vascular model with obstacles. Additionally, we will attempt to provide feedback regarding the microrobot’s position information calculated by the MDTS to the electromagnetic driving system. This will enable a real-time adjustment to the microrobot’s movement states, trajectory planning, and obstacle avoidance. Finally, our research will focus on leveraging deep learning techniques to enable the real-time detection and tracking of microrobots within the deep tissues of the human body. By inputting datasets obtained through X-ray or ultrasound imaging into a deep learning model, our goal is to establish a reliable foundation for the future application of deep learning in the medical field. This has the potential to revolutionize medical research and treatment, offering enhanced monitoring capabilities and improved outcomes for patients.

## Figures and Tables

**Table 1:** Comparison of experimental results of evaluation indicators before and after improved YOLOv5.

Index of Evaluation	E			Improved YOLOv5		
	1 mm	3 mm	Microrobot (2 mm)	1 mm	3 mm	Microrobot (2 mm)
P/%	89.70	93.71	90.20	91.03	95.81	91.70
R/%	89.73	90.21	92.60	90.30	92.33	94.30
mAP/%	89.00	95.70	94.50	91.90	96.80	96.20

## Data Availability

The original contributions presented in the study are included in the article/Supplementary Material, further inquiries can be directed to the corresponding author.

## Supplementary Materials

The following supporting information can be downloaded at: https://www.mdpi.com/article/10.3390/mi15060756/s1, Figure S1: An improved YOLOv5 network structure module. (a) The internal structure of the swing transformer module, (b) Structure framework introduction of the C3STR module, (c) Structure framework introduction of the ODConv module; Figure S2: The schematic diagram illustrates the monocular camera’s imaging principle. Here, D represents the actual distance from the microrobot to the camera’s optical center, f is the camera’s focal length, w denotes the actual size of the microrobot, and p corresponds to the pixel size of the microrobot on the imaging plane; Figure S3: A schematic diagram of the relationship between image coordinate system and pixel coordinate system; Figure S4: A schematic diagram of the principle of binocular imaging; Figure S5: The 2D and 3D vascular models for the experiment. (a) The parameter information of the 2D vascular model. (b) The parameter information of the 2D blood vessel model with thrombus. (c) The parameter information for the 3D vascular model. Video S1: Microrobot self-curling optical microscopy images and simulation images. Video S2: Real-time detection and tracking of two-dimensional motion of microrobot. Video S3: Real-time detection and tracking experiment of microrobot in obstacle channel. Video S4: Real-time detection and tracking of three-dimensional motion of microrobot.
