# Supplementary material for: Magnetic-Controlled Microrobot: Real-Time Detection and Tracking through Deep Learning Approaches
Source: Micromachines (Basel). 2024 Jun 5;15(6):756. doi: 10.3390/mi15060756 (PMC11205840; doi:10.3390/mi15060756)
Supplement: Supplementary file 1 [file micromachines-15-00756-s001.zip › Supplementary Information S1.pdf]

Supplementary Information

Magnetic-Controlled Microrobot: Real-Time Detection and Tracking through Deep Learning Approaches

Hao Li [1], Xin Yi [2], Zhaopeng Zhang [2] and Yuan Chen [1,*]

[1] Department of Mechatronics and Information Engineering, Shandong University at Weihai, Weihai 264209, China; yustlh@sdu.edu.cn
[2] Department of Mechanical Engineering, Yanbian University, Yanji 133002, China; yxin0912@gmail.com (X.Y.); rocman215@outlook.com (Z.Z.)
* Correspondence: cyzghysy@sdu.edu.cn

Table of Contents

Derivation formula for target tracking algorithm

Note1. Optimization of network structure

Note2. Schematic diagram of monocular camera imaging principle

Note3. Schematic diagram of the relationship between image coordinate system and pixel coordinate system

Note4. Schematic diagram of the principle of head up binocular imaging

Note5. Simulate human hepatic vein vascular model.

Supplementary Video 1-4

Derivation formula for target tracking algorithm

To convert the pixel coordinate system to the world coordinate system, we can use the following calculation formulas:

$$\begin{cases} f = (\Delta p \cdot D) / w \\ f = (|\boldsymbol{h}| \cdot D) / |\boldsymbol{H}| \end{cases} \tag{1}$$

By applying formula 1, we can determine the true distance of the target's motion in each frame and the accumulated true distance of the entire motion stage. The calculation formulas are as follows:

$$l_w = \frac{l_p \cdot w}{\Delta p} \tag{2}$$

$$L_w = \frac{L_p \cdot w}{\Delta p} \tag{3}$$

By calculating the true distance L_w for each frame using the above formulas, we can calculate the target's motion speed. The calculation formula is as follows:

$$v = \frac{l_w}{T} = l_w \cdot FPS \tag{4}$$

Where *FPS* represents the frame rate for camera video capture. The formula for calculating the world coordinates of the target center is:

$$|\boldsymbol{H}| = \frac{|\boldsymbol{h}| \cdot w}{\Delta p} \tag{5}$$

The formula for calculating the distance between the target center and the origin of the pixel coordinate system is:

$$|\boldsymbol{h}| = \left\| \begin{bmatrix} x_i \\ y_i \end{bmatrix} \right\| = \sqrt{x_i^2 + y_i^2} \tag{6}$$

Pixel coordinate system and Image coordinate system:

Both the pixel coordinate systems and image coordinate systems are established on the camera's imaging plane, differing in their coordinate origin and measurement units. Figure s3 illustrates the relationship between the two coordinate system. In the pixel coordinate system, the origin is situated in the upper-left corner of the entire imaging plane, and the unit of measurement is pixels, as depicted in the O-UV coordinate system in Figure s2. Conversely, the image coordinate system has its origin at the center of the imaging plane, with the unit of measurement being millimeters, as illustrated in the O-XY coordinate system in Figure s2. The conversion relationship between these two coordinate systems is outlined bellows:

$$\begin{bmatrix} u \\ v \\ 1 \end{bmatrix} = \begin{bmatrix} \frac{1}{\mathrm{d}x} & 0 & u_0 \\ 0 & \frac{1}{\mathrm{d}y} & v_0 \\ 0 & 0 & 1 \end{bmatrix} \begin{bmatrix} x \\ y \\ 1 \end{bmatrix} \tag{7}$$

In the formula, “dx” and “dy” denote the physical dimensions of each pixel in the image along the x-axis and y-axis directions, respectively, signifying that 1 pixel is equivalent to “dx” millimeters. The origin coordinates of the image coordinate system within the pixel coordinate system are designed as (u0, v0) .

Camera coordinate system and World coordinate system:

The origin of the camera coordinate system is situated at the optical center of the camera. The camera’s X axis and Y axes run parallel to the x and y axes of the image coordinate system, respectively. Meanwhile, the Z axis of the camera intersects the origin of the image coordinate system and stand perpendicular to the imaging plane. When a 3D point in space is projected onto the imaging plane, it occurs at the intersection of the ray and the imaging plane. This process, termed perspective projection, involves mapping a spatial point to the imaging plane. Utilizing the triangular geometric relationship, the transformation between the image coordinate system and the camera coordinate system can be derived as follows:

$$\begin{cases} x = \dfrac{f \cdot X_c}{Z_c} \\ y = \dfrac{f \cdot Y_c}{Z_c} \end{cases} \tag{8}$$

In the formula, f denotes the camera's focal length in millimeters, and (x, y) signifies the coordinates of the spatial point mapped onto the imaging plane within the image coordinate system. The utilization of homogeneous coordinates formulates expression as follows:

$$Z_c \begin{bmatrix} x \\ y \\ 1 \end{bmatrix} = \begin{bmatrix} f & 0 & 0 & 0 \\ 0 & f & 0 & 0 \\ 0 & 0 & 1 & 0 \end{bmatrix} \begin{bmatrix} X_c \\ Y_c \\ Z_c \\ 1 \end{bmatrix} \tag{9}$$

In the real-world scenario, acquiring the precise position of a spatial point necessitated the establishment of a world coordinate system. Once defined, this world coordinate system uniquely represents the spatial point’s three-dimensional coordinates (X, Y, Z) . Simultaneously, this spatial point is characterized by an exclusive set of three-dimensional coordinates (Xc, Yc, Zc) within the camera coordinate system. The transformation relationship detailing the transition between the spatial point’s

coordinates in the world coordinate system and the camera coordinate system is expressed as follows:

$$\begin{bmatrix} X_c \\ Y_c \\ Z_c \\ 1 \end{bmatrix} = \begin{bmatrix} R & t \\ 0^T & 1 \end{bmatrix} \begin{bmatrix} X \\ Y \\ Z \\ 1 \end{bmatrix} \tag{10}$$

In the formula, R denotes the of 3×3 rotation matrix, and t signifies the 3×1 translation vector. Specifically, $0^T=(0,0,0)^T$.

In summary, the transformation linking three-dimensional points (X, Y, Z) in the world coordinate system to (u_0, v_0) in the pixel coordinate system is represented as follows:

$$Z_c \begin{bmatrix} u \\ v \\ 1 \end{bmatrix} = \begin{bmatrix} \frac{1}{\mathrm{d}x} & 0 & u_0 \\ 0 & \frac{1}{\mathrm{d}y} & v_0 \\ 0 & 0 & 1 \end{bmatrix} \begin{bmatrix} f & 0 & 0 & 0 \\ 0 & f & 0 & 0 \\ 0 & 0 & 1 & 0 \end{bmatrix} \begin{bmatrix} R & t \\ 0^T & 1 \end{bmatrix} \begin{bmatrix} X \\ Y \\ Z \\ 1 \end{bmatrix} = K \cdot \begin{bmatrix} R & t \\ 0^T & 1 \end{bmatrix} \begin{bmatrix} X \\ Y \\ Z \\ 1 \end{bmatrix} \tag{11}$$

In the equation, K represents the camera's internal parameter matrix, and it is the extrinsic matrix of the camera

1. Optimization of network structure

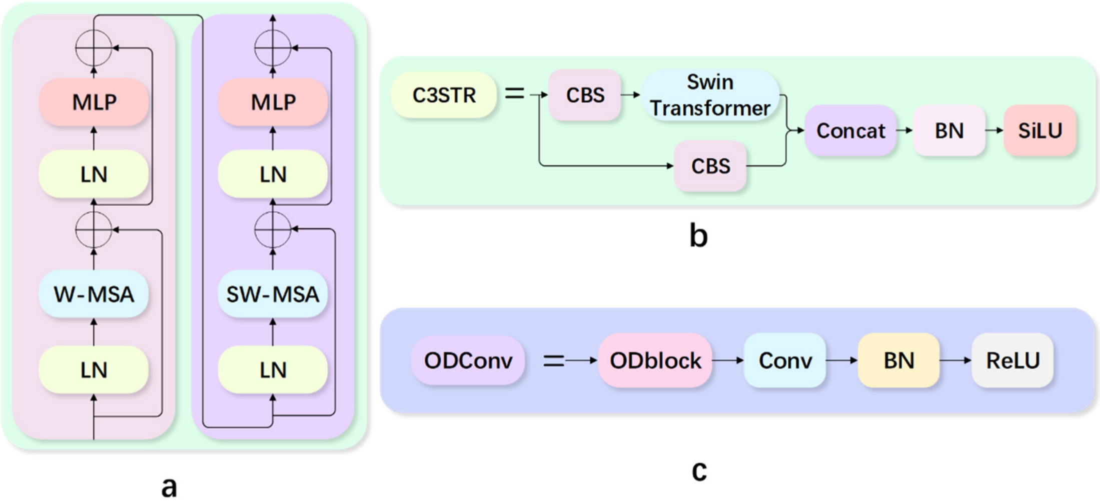

Fig.S1. Improved YOLOv5 network structure module. a) Internal structure of the swing transformer module, b) Structure framework introduction of the C3STR module, c) Structure framework introduction of the ODConv module.

2. Schematic diagram of monocular camera imaging principle

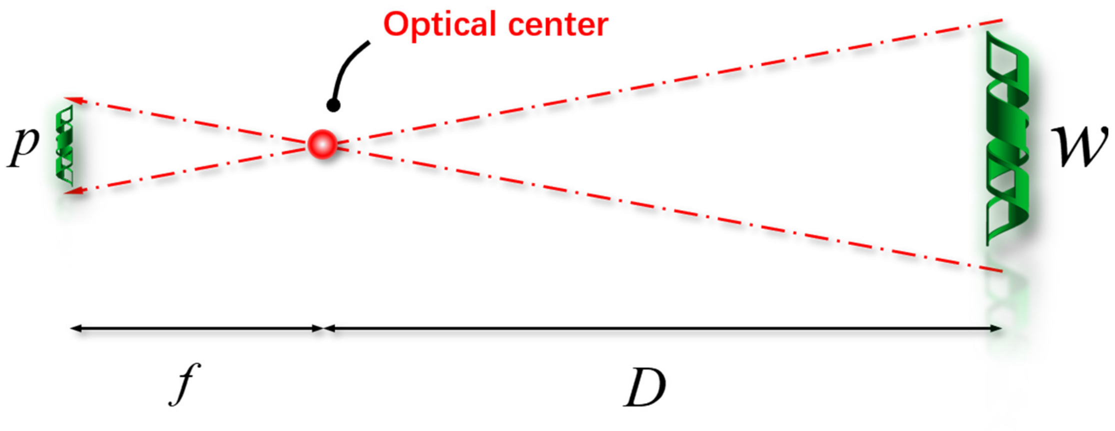

Fig.S2. The schematic diagram illustrates the monocular camera's imaging principle. Here, D represent the actual distance from the microrobot to the camera's optical center, f is the camera's focal length, w denotes the actual size of the microrobot, and p corresponds to the pixel size of the microrobot on the imaging plane.

3. Schematic diagram of the relationship between image coordinate system and pixel coordinate system

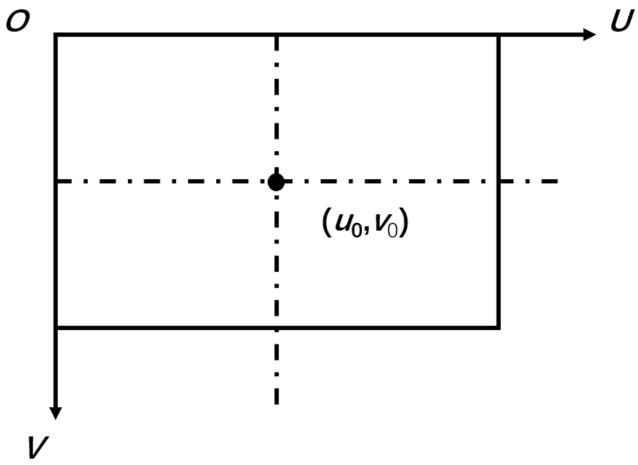

Fig.S3. Schematic diagram of the relationship between image coordinate system and pixel coordinate system.

4. Schematic diagram of the principle of head up binocular imaging

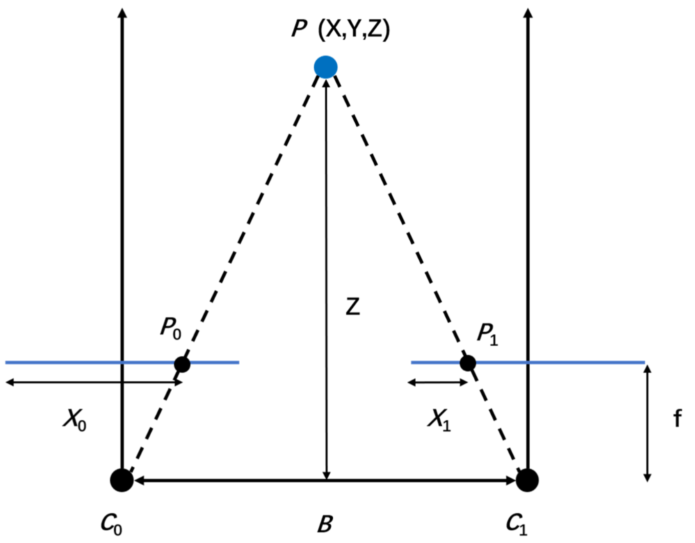

Fig.S4. Schematic diagram of the principle of binocular imaging.

5. Simulate human hepatic vein vascular model.

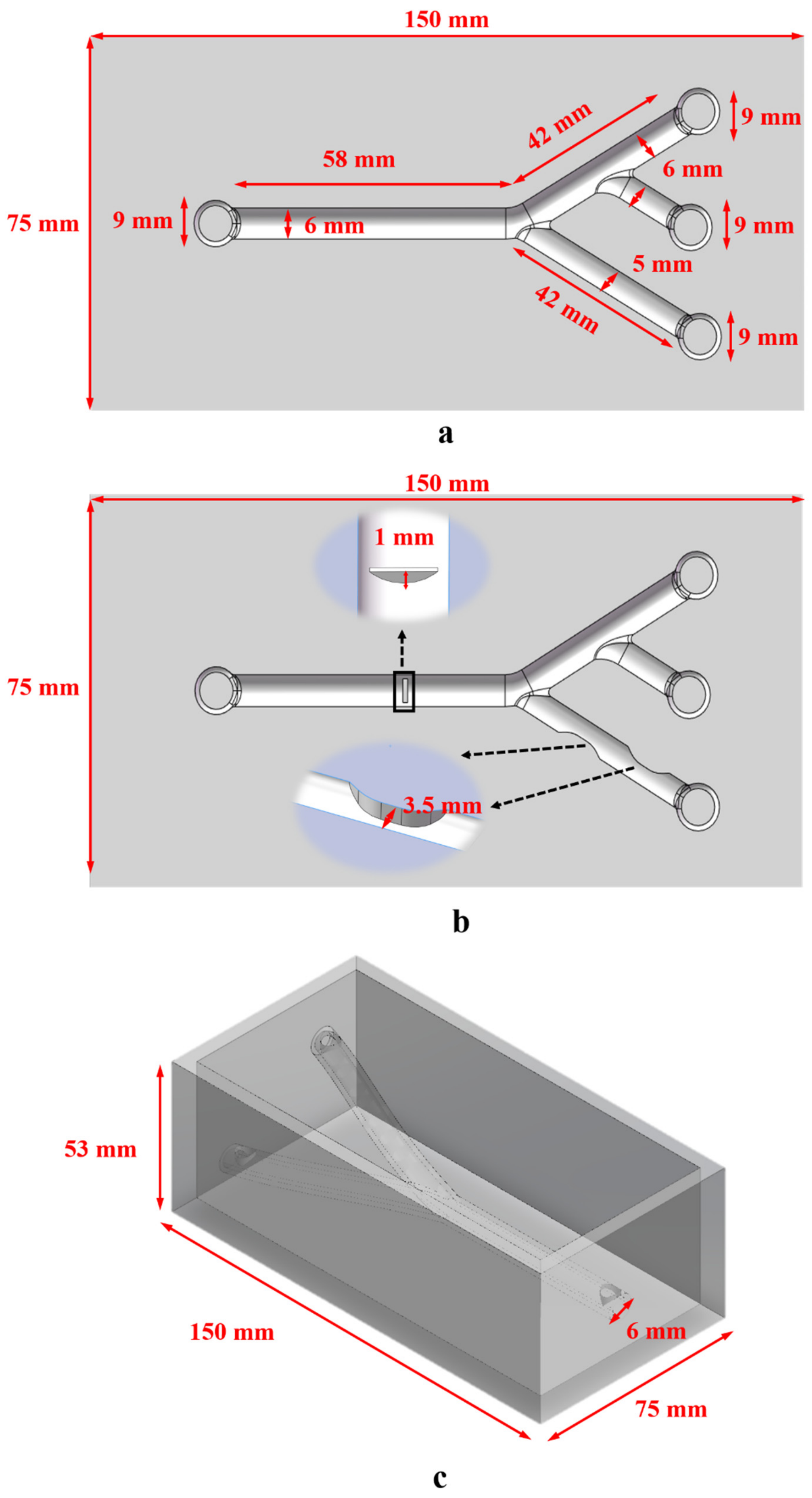

Fig.S5. The 2D and 3D vascular models for the experiment. a) parameter information of the 2D vascular model. b) parameter information of the 2D blood vessel model with thrombus. c) parameter information for the 3D vascular model.
