# Supplementary figures and images for: Magnetic-Controlled Microrobot: Real-Time Detection and Tracking through Deep Learning Approaches
Source: Micromachines (Basel). 2024 Jun 5;15(6):756. doi: 10.3390/mi15060756 (PMC11205840; doi:10.3390/mi15060756)

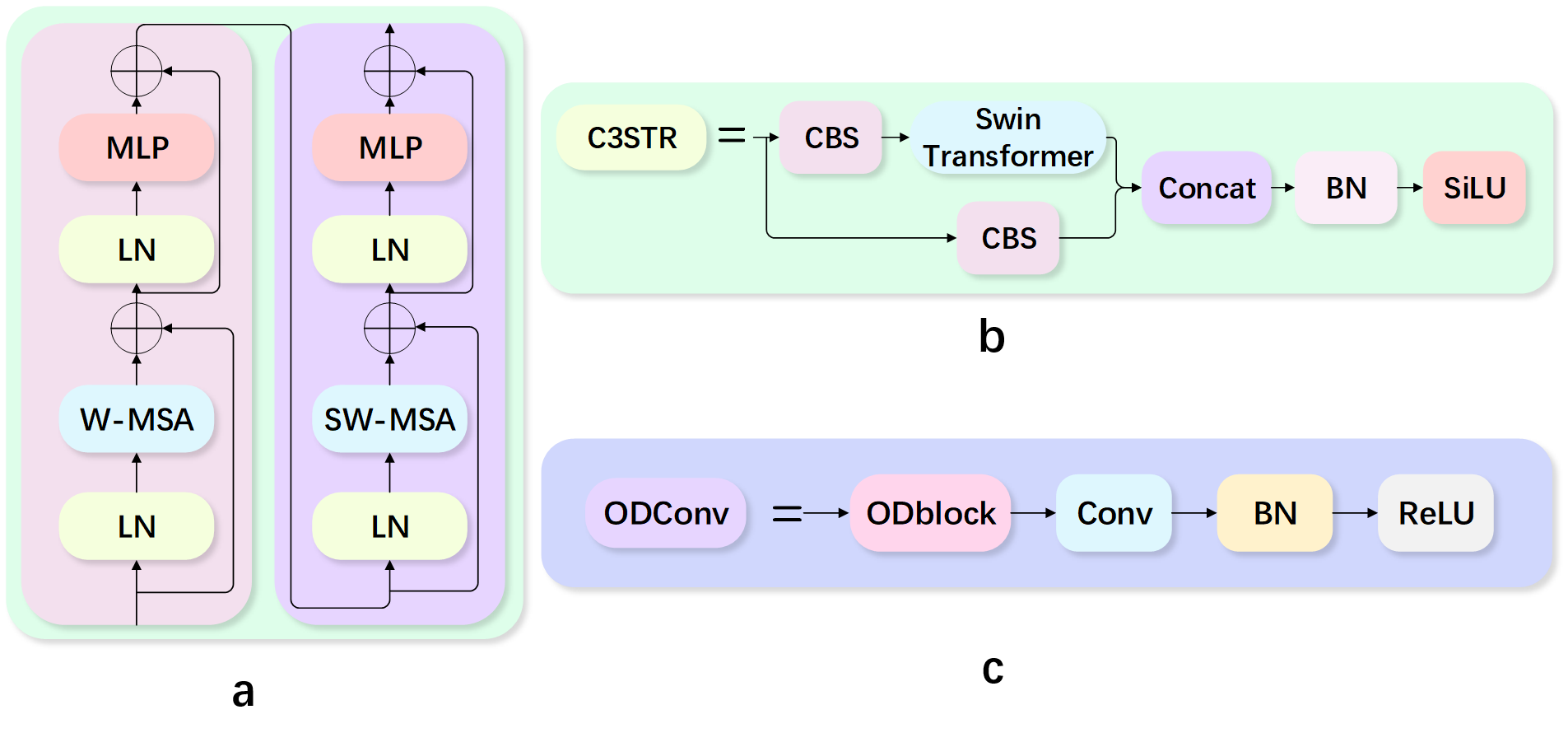

Supplement: Supplementary file 1 [file micromachines-15-00756-s001.zip › figs1.png]

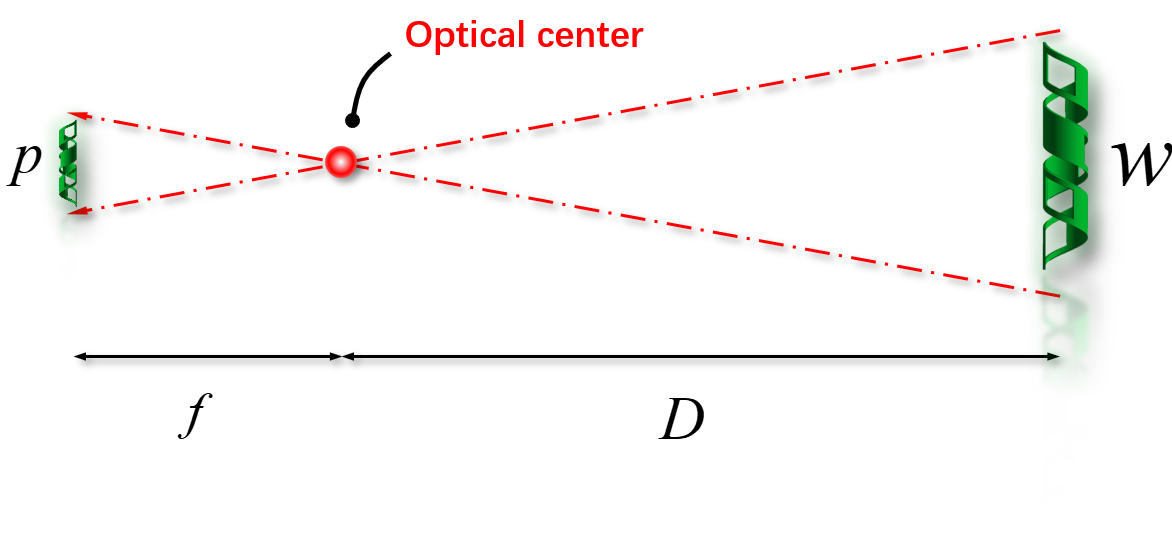

Supplement: Supplementary file 1 [file micromachines-15-00756-s001.zip › figs2.png]

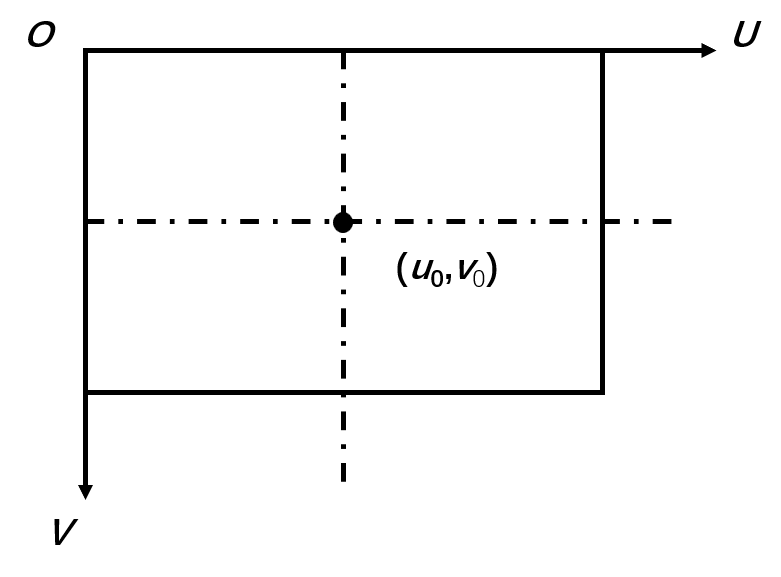

Supplement: Supplementary file 1 [file micromachines-15-00756-s001.zip › figs3.png]

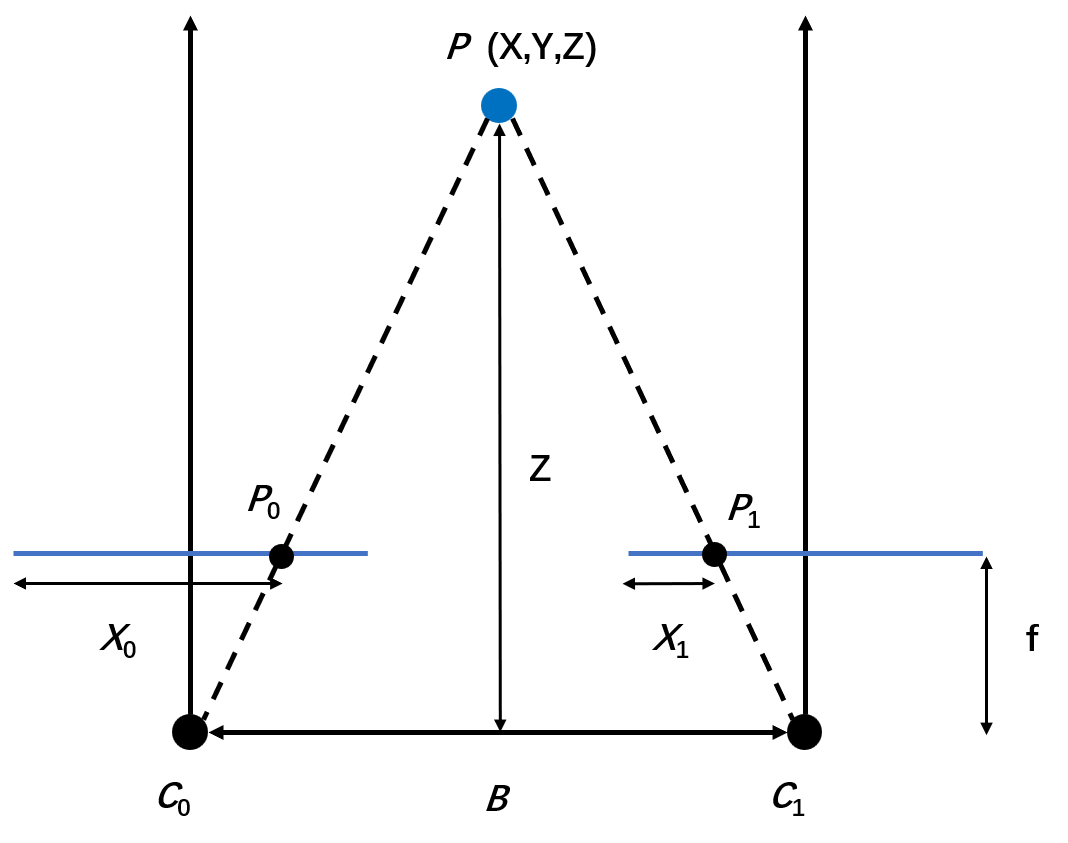

Supplement: Supplementary file 1 [file micromachines-15-00756-s001.zip › figs4.png]

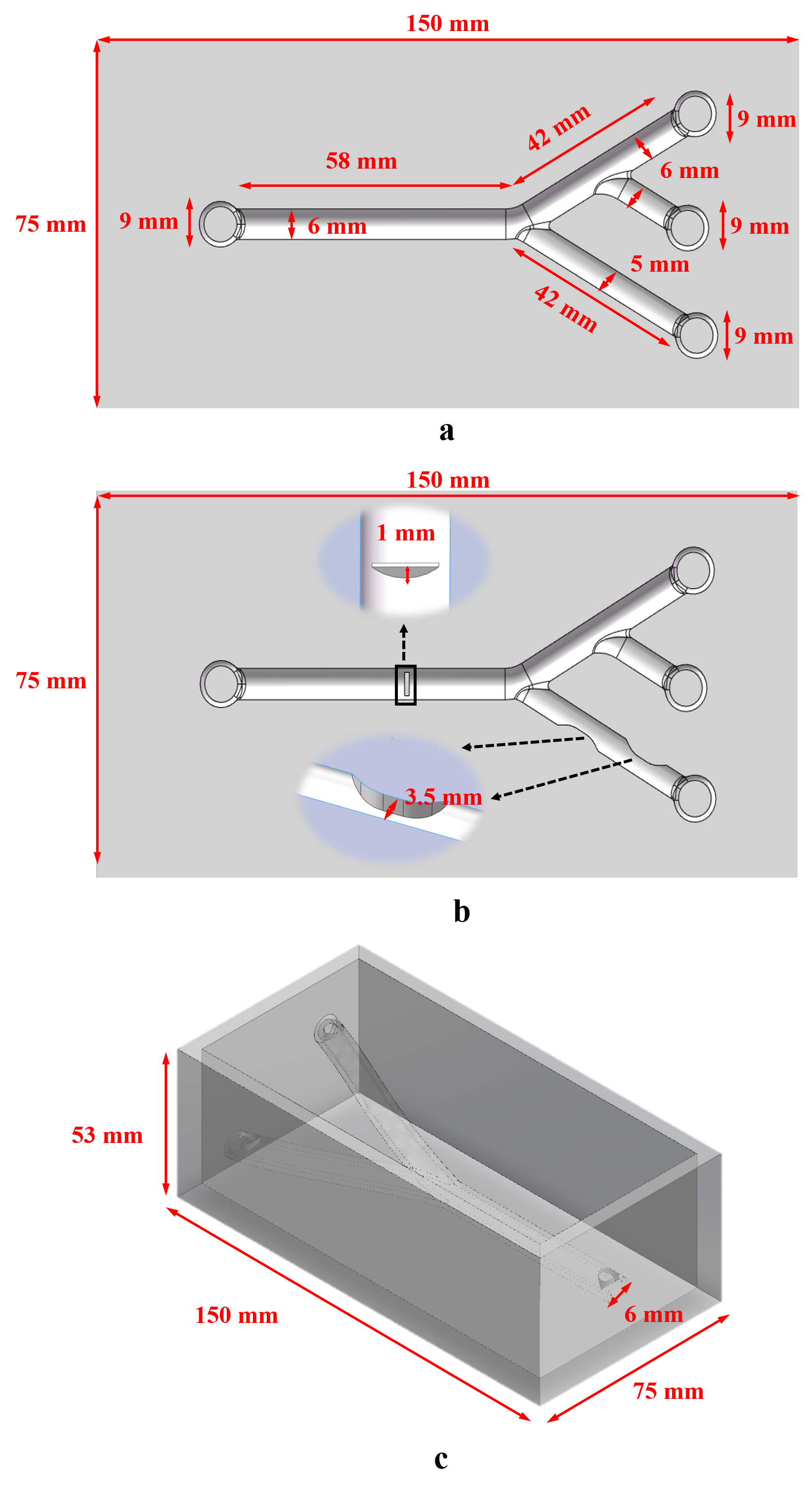

Supplement: Supplementary file 1 [file micromachines-15-00756-s001.zip › figs5.png]
